# A Silent Threat: Acute Respiratory Failure and Os Odontoideum in a Child With Down’s Syndrome

**DOI:** 10.7759/cureus.90227

**Published:** 2025-08-16

**Authors:** Dritan Pasku, Rawan Masarwa, Amit Kumar Bhanushali, Elie Najjar, Weronika Nocun, Daniel D'Aquino

**Affiliations:** 1 Orthopaedics, The Centre for Spinal Studies and Surgery, Queen's Medical Centre, University Hospital NHS Trust, Nottingham, GBR; 2 Surgery, The Centre for Spinal Studies and Surgery, Queen's Medical Centre, University Hospital NHS Trust, Nottingham, GBR; 3 School of Medicine, University of Nottingham, Nottingham, GBR; 4 Internal Medicine, The Centre for Spinal Studies and Surgery, Queen's Medical Centre, University Hospital NHS Trust, Nottingham, GBR

**Keywords:** acute respiratory distress, atlantoaxial instability, down's syndrome, non-traumatic os odontoideum, os odontoideum, respiratory failure

## Abstract

We report the case of a 6-year-old boy with Down syndrome who developed acute neurological deterioration and respiratory distress secondary to non-traumatic os odontoideum. Although the patient had a history of global developmental delay, he exhibited atypical and progressive gross motor regression, prompting further evaluation. Cervical spine MRI revealed severe cranio-cervical junction stenosis with significant upper cervical cord compression and myelomalacia. CT angiography confirmed marked atlantoaxial dislocation due to an anteriorly displaced os odontoideum and a hypoplastic odontoid peg. The patient was placed on the priority surgical list and discharged home.

Three weeks post-discharge, he presented to the ED in respiratory arrest and was admitted to the paediatric ICU (PICU). Urgent occipito-cervical (C0-C5) fusion and C1 posterior arch decompression surgery were performed to maximize the possibility of respiratory improvement. Postoperatively, he required prolonged supportive care, including supplemental oxygen and halo vest immobilisation. He was discharged nearly three months later with full neurological recovery and stable respiratory function. This case underscores the critical importance of early recognition of cervical spine instability in patients with Down syndrome and os odontoideum to prevent severe spinal cord compression and life-threatening complications, while also highlighting the potential for favorable outcomes with timely surgical intervention.

## Introduction

Os odontoideum (OO), a rare but critical cause of cervical spine instability, poses significant challenges in patients with Down’s syndrome (DS), who are already predisposed to craniovertebral junction (CVJ) anomalies. The term OO refers to hypoplasia of the odontoid process, with a detached, smooth-margined ossicle located above the superior facets of the axis (C2) [1,2].

Down syndrome is the most common chromosomal disorder, occurring in approximately 1 in 660 live births. One-third of DS cases involve the CVJ, leading to an increased risk of atlantoaxial instability (AAI) [3]. OO is a recognized cause of AAI, affecting approximately 10-20% of individuals with Down syndrome [4]. Although the pathogenesis of OO remains debated, both congenital and acquired (traumatic) mechanisms have been proposed. While a congenital origin has historically been suggested, recent evidence supports the acquired (traumatic) theory, which posits that blood supply deficiency and reduced bone mass at the base of the odontoid make these children, often with intellectual disabilities, more susceptible to undetected fractures from low-grade trauma [5,6].

In DS patients, additional factors, such as low bone mineral density, transverse ligament laxity, low muscle tone, and excessive joint flexibility, further contribute to the development of AAI [1,2]. AAI in patients with OO manifests more commonly in childhood or adolescence, with symptoms ranging from abrupt onset following minor trauma to gradual neurological deterioration over several years [1,3].

In this report, we present the case of a 6-year-old boy with DS and non-traumatic OO, who developed acute neurological compromise and respiratory distress, highlighting the importance of early recognition and surgical intervention in such cases.

## Case presentation

Initial workup

A 6-year-old male with DS and non-traumatic os odontoideum (OO) had been under regular pediatric follow-up due to delayed independent walking, speech delays, and overall gross motor regression. The patient exhibited global developmental delay, achieving head control at approximately 5 months and sitting without support by 12 months. By the age of 3.5 years, he was able to pull to stand and cruise along furniture, but independent walking had not yet been achieved. At age 4, his progress plateaued, and over the following year, he demonstrated regression in gross motor abilities, including increased difficulty with standing and decreased mobility. This prompted further evaluation by the pediatric DS team, who noted the atypical nature of the regression even within the context of DS.

Over the previous two years before presentation, the patient underwent investigations, including X-rays and ultrasound scans (USS), to assess for skeletal anomalies of the pelvis and lower limbs, which were unremarkable. The pediatric DS team subsequently decided to perform an MRI of the head and spine to rule out cerebral, cerebellar, and/or cervical spine instability.

**Figure 1:**
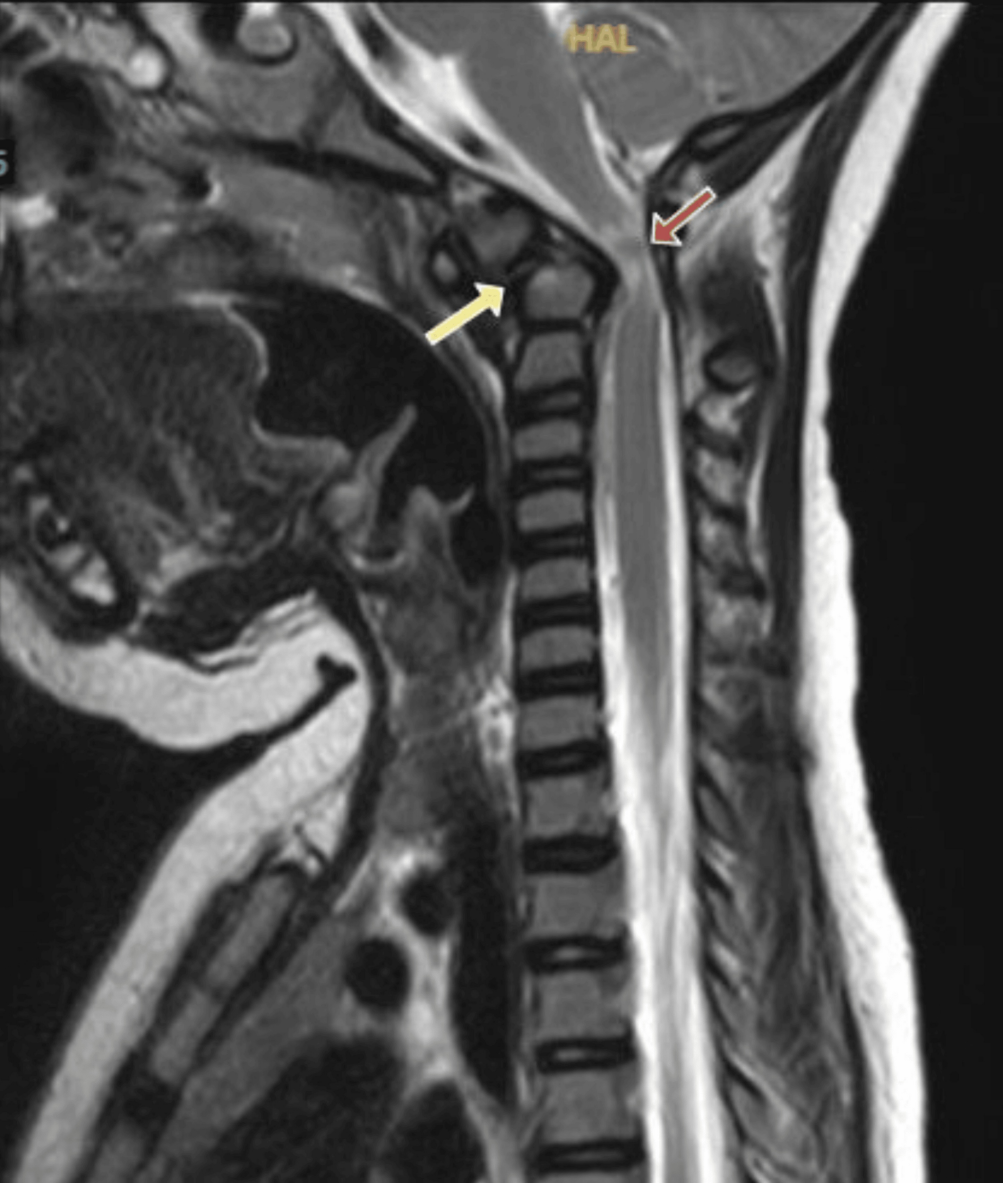
Sagittal T2-weighted MRI of the cervical spine showing cervicomedullary cord compression (red arrow) secondary to anterior subluxation of os odontoideum (yellow arrow).

MRI of the cervical spine revealed severe stenosis at the cranio-cervical junction, with upper cervical cord compression and significant myelomalacia (Figure 1).

**Figure 2:**
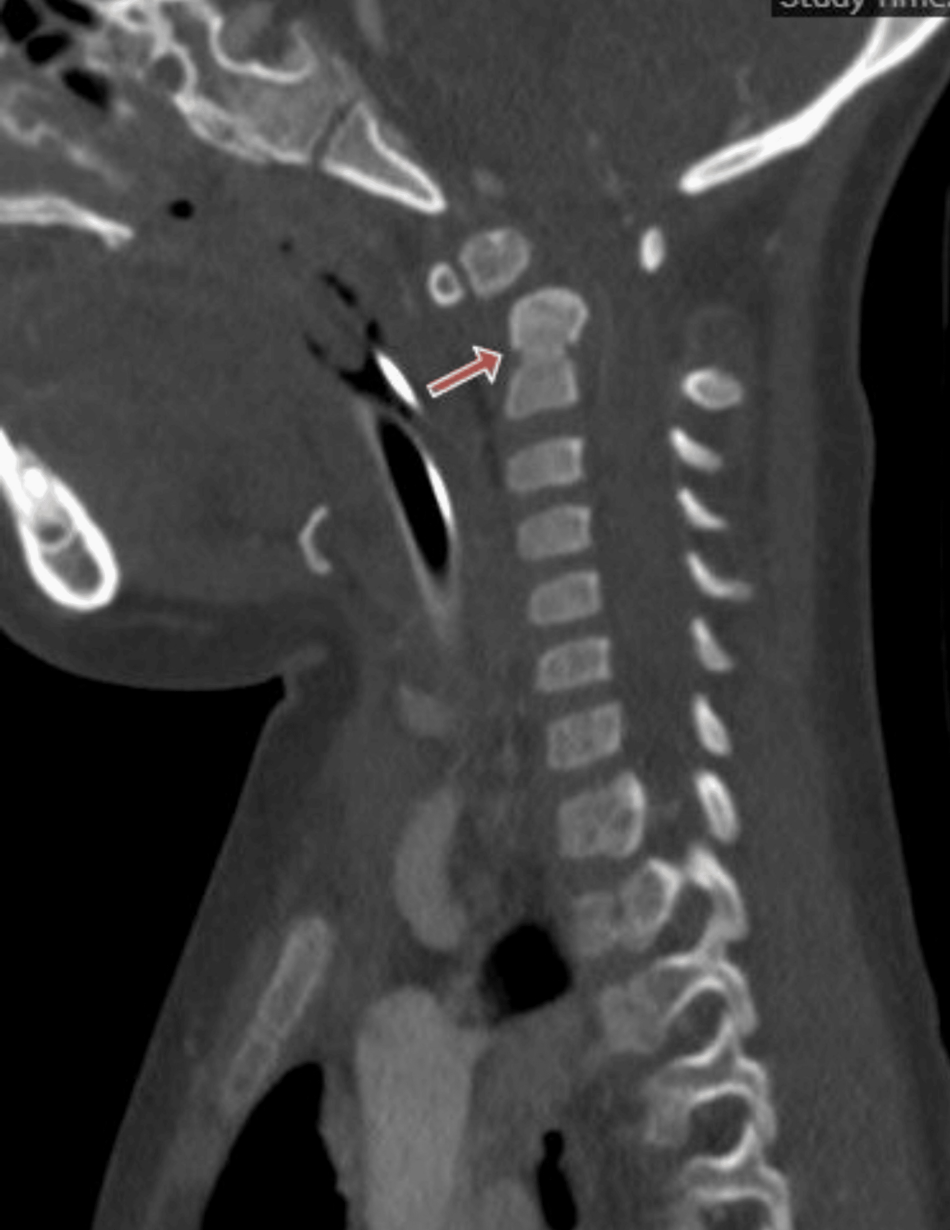
Sagittal sequence of the CT angiography showing os odontoideum with atlantoaxial subluxation (red arrow).

A CT angiography was performed and showed a marked atlantoaxial dislocation due to an anteriorly dislocated os odontoideum and a hypoplastic odontoid peg (Figure 2).

The atlanto-dens interval (ADI) measured 7 mm, while the rest of the spinal cord from the distal cervical to thoracic segments was normal. Brain MRI showed immature hippocampi, a common feature in DS.

The patient was referred to our spinal team based on these findings. On examination, the patient had satisfactory movements in all four limbs; however, there was increased tone in the lower limbs and positive bilateral ankle clonus. There were no signs of systemic illness or respiratory compromise. An ASPEN collar was applied to stabilize the neck and prevent further deterioration. Following a multidisciplinary team (MDT) discussion, surgical intervention was recommended, with consensus on occipito-cervical fusion due to the concerning anatomical and clinical presentation. The patient was discharged home with detailed information provided to the parents, and he was placed on the priority surgical list.

In March 2023, three weeks post-discharge, the patient presented to the emergency department in a lethargic state, with abnormal breathing. According to the parents, he had developed a cough and semi-solid diarrhea over the previous three days. There was no history of recent trauma. On assessment in the resuscitation unit, the patient went into respiratory arrest, with oxygen saturation levels of 58% on room air and 73% on high-flow oxygen. His Glasgow Coma Scale (GCS) score was 3/15, heart rate (HR) was 120 bpm, and blood pressure (BP) was 114/70 mmHg.

**Figure 3:**
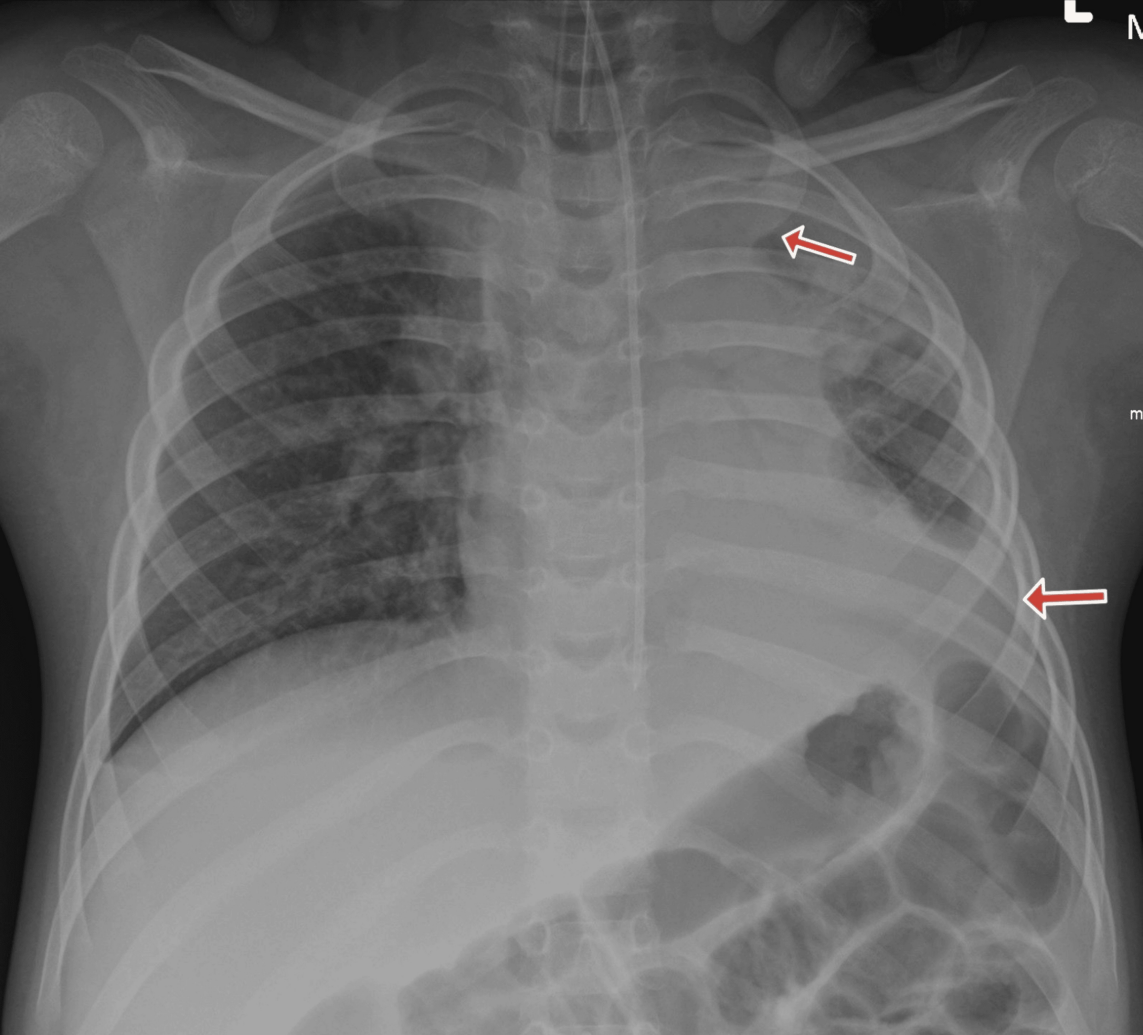
Chest X-ray showing consolidations in both upper lobes, more marked on the left (highlighted with red arrows).

The patient was intubated, ventilated, and placed on a sepsis management protocol. A chest X-ray showed mild lung consolidation, which did not fully account for the respiratory arrest (Figure 3).

A repeat MRI of the head and spine showed a similar appearance of cord edema as in the previous MRI. The patient was admitted to the PICU, and cervical traction was applied with an initial weight of 2 lbs, gradually increased over time. Additionally, no vertebral artery anomaly was noted, which was confirmed and double-checked from the CT angiography performed previously. Given the patient’s clinical deterioration and MRI findings, urgent surgical intervention was decided after an extended MDT discussion in the presence of pediatric intensivists and anesthetists.

Surgery

**Figure 4:**
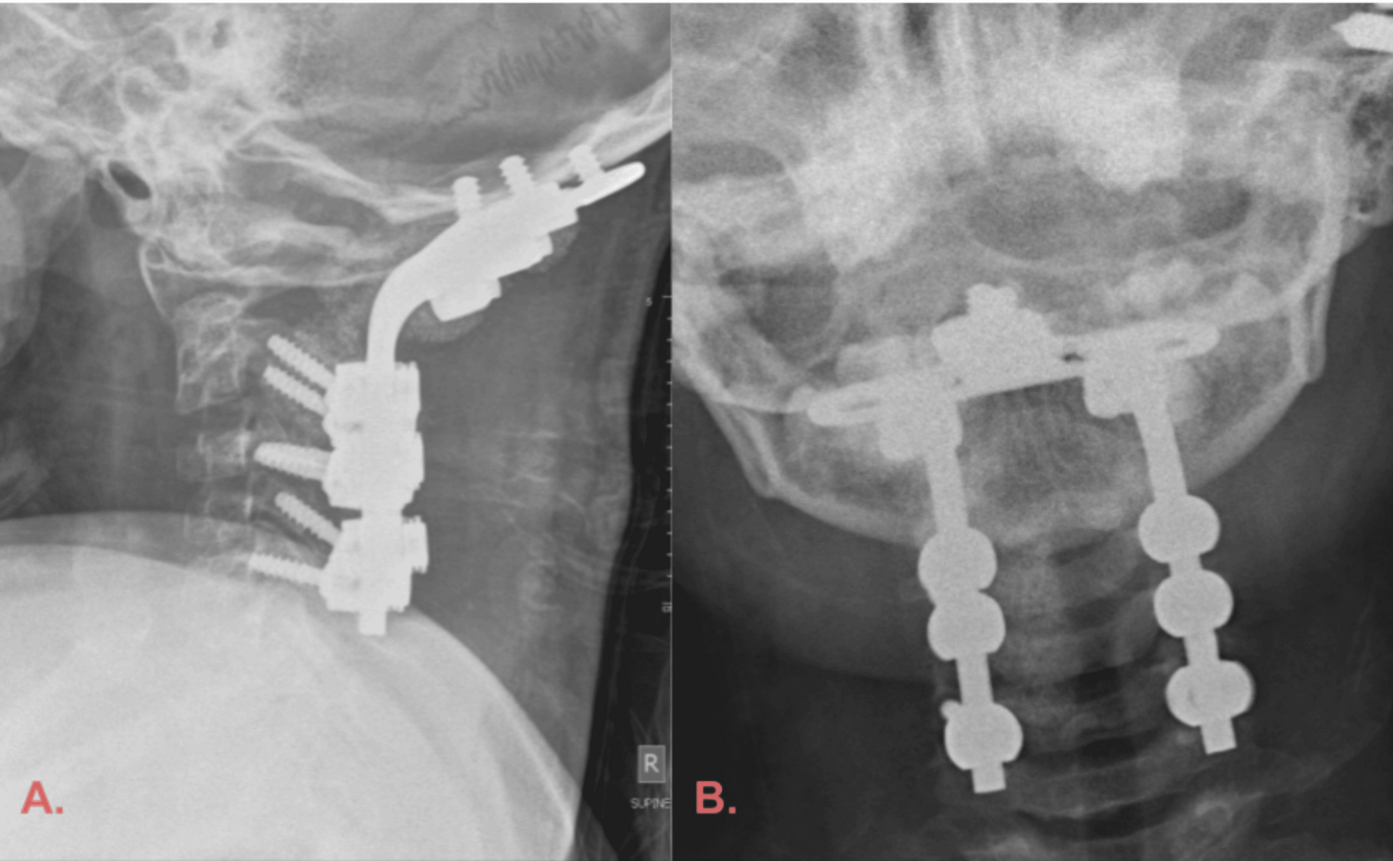
(A) Lateral; (B) Anteroposterior cervical spine X-ray showing the instrumented occipito-cervical fixation.

Within three days of admission, we performed occipito-cervical C0-C5 fusion and C1 posterior arch decompression (Figures 4a-4b).

Autologous and synthetic demineralized bone grafts were used for fusion. There were no intraoperative complications, and the estimated blood loss was 100 ml. A rigid cervical collar was applied postoperatively to protect the instrumentation. The patient remained intubated for five days and required extended PICU care due to slow improvement in respiratory function.

Recovery

Three weeks post-surgery, a halo vest was applied for additional stability, as the patient had become increasingly active and playful. He tolerated the halo vest well, and it was removed after two months. Neurological improvement was gradual during the first three weeks, with complete recovery by week four. His respiratory function also improved steadily, with a reduced need for nasal oxygen over time. By the end of the third week, he was breathing independently on room air. The patient was discharged nearly three months after surgery, with full neurological recovery and stable respiratory function.

## Discussion

This case represents a unique presentation of respiratory arrest secondary to severe cord compression in a patient with DS and OO, a rarely reported complication. Trisomy 21, or DS, is the most common chromosomal disorder, affecting multiple organ systems, and is frequently associated with orthopedic abnormalities, particularly of the spine. Besides cognitive impairments, DS is known for gross motor delays due to ligamentous laxity and poor muscle tone, which contribute to overall balance issues and walking ability [7].

Spitzer et al. first reported cervical spine instability in association with DS in 1961 [8]. Radiographically, AAI has been reported in 10% to 30% of patients with DS, though only approximately 1.5% are estimated to require surgical stabilization [9].

Cord compression at the C1-C2 level can present with a range of symptoms, from asymptomatic cases to tetraparesis and severe cardiorespiratory compromise [10]. Most patients experience long-standing symptoms before deterioration, though acute neurological decline has been reported in some cases [11,12].

AAI is the primary cause of cord compression and symptomatic cervical myelopathy in DS, occurring in approximately 8.5% of cases, though this may often be underdiagnosed [13]. Although most studies have focused on AAI, there is limited literature defining abnormalities of the atlanto-occipital joint [12,14].

OO is the most significant cervical spine anomaly in patients with DS, contributing directly to AAI. Various cranio-cervical osseous abnormalities, such as hypoplastic atlas, congenitally abnormal C2, bifid odontoid process, and third condyle, have been reported in these patients, but OO has been specifically associated with a greater risk of instability requiring surgical stabilization [12,15].

Radiographically, the most critical findings in OO are an atlanto-dens interval (ADI) of greater than 5 mm, measured on X-ray, and a space available for the cord (SAC) of less than 14 mm, measured on CT scan [12]. However, radiographic measures alone should not dictate surgical intervention. Patients with an SAC of less than 14 mm or an ADI greater than 9.9 mm are more likely to show symptoms of spinal cord compression and require close clinical evaluation and follow-up. A low threshold should be maintained for performing cervical MRI to rule out myelomalacia [12,14,15]. Brockmeyer has strongly recommended surgical fusion in cases where OO is present since the 1990s [15].

In this case, the patient presented with an ADI of 7.6 mm and an SAC of 8.0 mm. However, these measurements are often unreliable in OO cases complicated by dislocation. Given the clinical scenario, the patient had a clear indication for reduction and fusion surgery. Unfortunately, while awaiting surgery, the patient developed a lower respiratory tract infection. Although the clinical and radiological findings were consistent with an uncomplicated infection, the patient unexpectedly suffered respiratory arrest. This underscores the need to interpret imaging thresholds cautiously within the context of the overall clinical scenario and underlying pathology.

We speculate that the changes in the patient’s spinal cord were already advanced, and he was unable to cope with the infection, leading to severe acute respiratory failure. This case underscores the importance of balancing surgical timing when infection is present, as further delay could have resulted in irreversible neurological damage. After thorough discussion, surgery was performed to give the patient the best chance of recovery, despite the infection. The patient required prolonged respiratory support in the PICU and was discharged to the ward almost two months after surgery.

To our knowledge, this is the first reported case of respiratory arrest in a patient with DS and OO, highlighting the critical need for early recognition and prompt management in such cases.

## Conclusions

In children with DS who exhibit motor development delays beyond the expected range, early screening for atlantoaxial dislocation and cervical cord compression may be warranted. When radiological evidence of instability is present alongside clinical suspicion, it is important to provide comprehensive counselling to parents regarding the potential risks and benefits of treatment. Early surgical intervention should be prioritized to prevent critical cord compression, which can lead to severe neurological and cardiorespiratory complications.

This case highlights the importance of vigilance in early detection and the necessity of a multidisciplinary approach to optimize treatment outcomes and reduce life-threatening risks. Timely surgery not only helps prevent neurological decline but also significantly improves overall prognosis.
